# Pigmented Malignant Lesion of Central Arch Gingiva of Mandible

**DOI:** 10.7759/cureus.12210

**Published:** 2020-12-21

**Authors:** Puja Khanna, Jitendra S Nigam, Tarun Kumar, Jagjit Pandey

**Affiliations:** 1 Pathology and Lab Medicine, All India Institute of Medical Sciences, Patna, IND; 2 Surgical Oncology, All India Institute of Medical Sciences, Patna, IND

**Keywords:** gingiva, oral cavity, malignant melanoma

## Abstract

Primary oral malignant melanoma (OMM) is a rare malignant lesion with a relatively poor prognosis. The clinical presentation may be nodular, macular, or mixed type, with or without pigmentation. The pigmented lesions in the oral cavity may be either melanocytic or non-melanocytic and neoplastic or non-neoplastic lesions. For all the pigmented lesions of the oral cavity, clinicians must consider OMM as a differential diagnosis. Hence, a histopathological examination is required. Herein, we report a case of pigmented growth over the central arch and gingival mucosa, which turned out to be an OMM.

## Introduction

Melanocytes are more commonly found in the skin; however, they are also allocated along the mucosal membrane of the anorectal region, cervix, vagina, nasal cavity, paranasal sinuses, larynx, esophagus, and oral cavity. The most common site for oral mucosal melanoma (OMM) is the upper jaw. Mucosal malignant melanoma constitutes approximately <1% of all melanoma, out of which 10% occurs in the head and neck region [1]. WHO classification of head and neck tumors 2017 enlists oral mucosal melanoma as a separate entity [2]. Exposure to formaldehyde fumes and smoking may be considered a risk factor for OMM; however, there is no association with oral hygiene, damaged teeth, dental caries, prosthetics, and other physical, chemical, or thermal irritants found [3]. The OMM shows a slight male predominance with a median age of 55-56 years [2]. The prognosis of OMM is relatively poor: five-year survival is between 10% to 30% [1, 3]. OMM's clinical presentation may be nodular, macular, or mixed type, with or without pigmentation [4]. The oral cavity's pigmented lesions vary from reactional to neoplastic processes and may be either a melanocytic or a non-melanocytic in origin [2, 5]. The melanocytic lesions comprise of smoker's melanosis, racial pigmentation, post-inflammatory pigmentation, oral nevi, melanotic macule, melanoacanthoma, OMM, the melanotic neuroectodermal tumor of infancy, and systemic diseases such as Addison’s disease, Laugier-Hunziker syndrome, Cushing syndrome, Kaposi sarcoma, and Peutz-Jeghers syndrome. Non-melanocytic lesions encompass heavy metal pigmentation, amalgam tattoo, lingua villosa nigra, and exogenous pigmentations [1, 2, 4, 5]. All the pigmented lesions of the oral cavity do not have disease-specific clinical features but need distinct management. Hence, the histopathological examination is required to rule out an early stage of OMM [4, 5]. Herein, we report a case of pigmented growth over the central arch and gingival mucosa, which turned out to be an OMM.

## Case presentation

**Figure 1:**
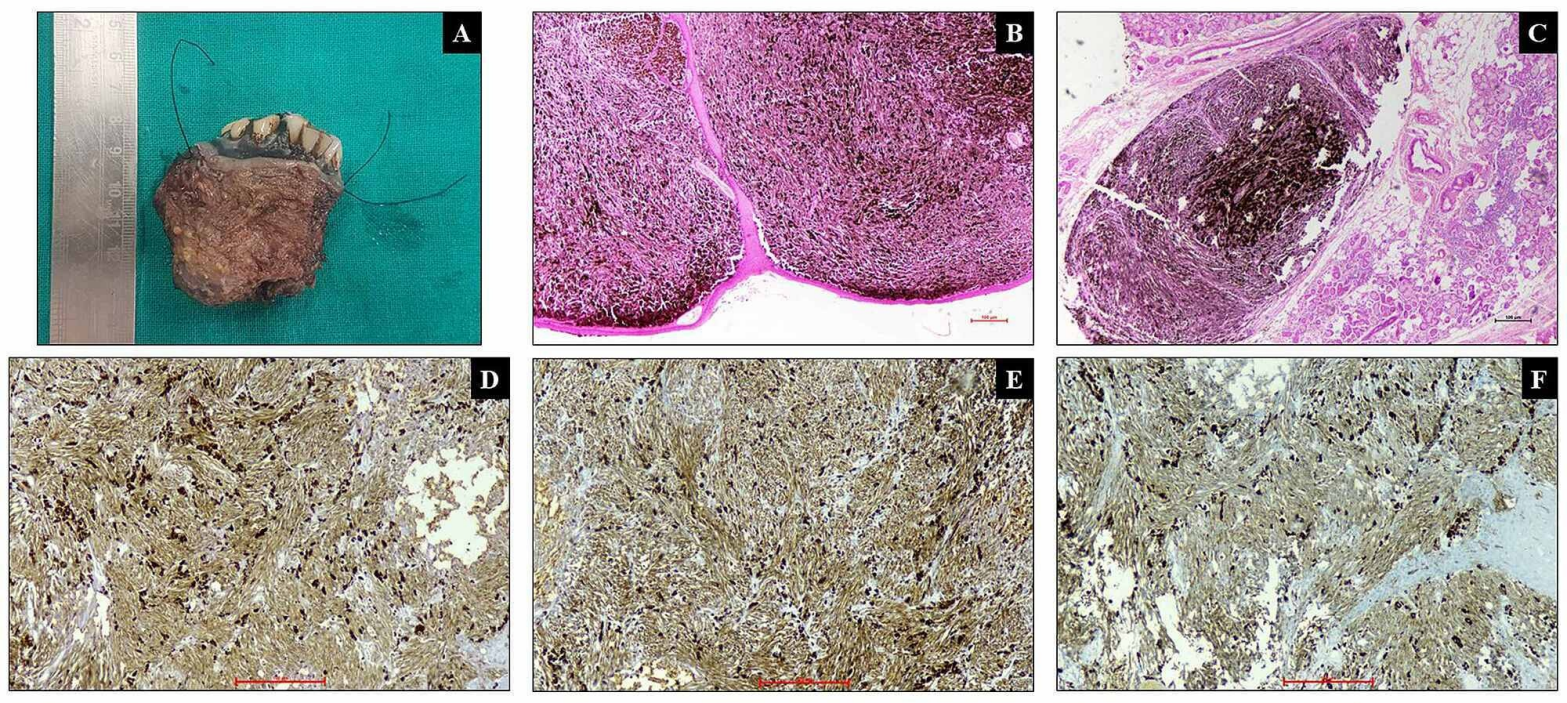
Gross and microscopy A. Gross: pigmented lesion of gingiva along with teeth. B. Poorly circumscribed pigmented tumor with overlying mucosa (H&E x40). C. Pigmented tumor cells with adjacent seromucinous glands (H&E x40). D. HMB- 45: diffuse, cytoplasmic positivity in tumor cells (x100). E. CD117: diffuse, cytoplasmic positivity in tumor cells (x100). F. BRAF: diffuse, cytoplasmic positivity in tumor cells (x100).

A 50-year-old male patient presented with the complaint of a painless, blackish growth measuring 2x1.5 cm over the central arch of the mandible for the last six months. No pigmented cutaneous lesions were found. A punch biopsy revealed a diagnosis suggestive of OMM. The computed tomography of the face and neck revealed a lytic lesion measuring 1.9x1.4 cm in the central arch of the mandible. The multiple subcentimetric lymph nodes were seen bilaterally, and the metastatic submental node (1.7x1.5 cm) was also noted. After the radiological evaluation for the extent of the lesion, wide local excision with modified radical neck dissection was done. Grossly, a blackish lesion measuring 3.5x3x3 cm involving the gingivobuccal sulcus was identified. Microscopy revealed a tumor arranged in diffuse sheets and nests composed of oval to spindle cells showing moderate nuclear pleomorphism with vesicular to hyperchromatic nuclei and prominent eosinophilic nucleoli. The intracellular and extracellular pigment was also noted. The tumor cells were immunoreactive for HMB-45, CD117, and BRAF while negative for pan-cytokeratin (Figure 1).

All the margins of resection were free of tumor. Lymphovascular emboli were not identified. Out of the eighty-two lymph nodes examined, two showed metastasis with perinodal extension. The final diagnosis of oral mucosal malignant melanoma was rendered with stage II histopathological microstaging. Postoperative radiotherapy was given. No chemotherapy was given. The patient did not have a recurrence or locoregional metastasis after six months of follow-up.

## Discussion

Mucosal melanoma was first described by Weber in 1856 [6]. The primary incidence of OMM is higher in Asians and African ethnicities, as these races have a high prevalence of melanin pigmentation in the oral mucosa. Both palate and maxillary gingiva constitute approximately 80% cases of OMM with peak age between 65 to 79 years [1, 7]. Tavares et al. studied pigmented lesions in the oral cavity. They observed that the maximum patient is middle-aged female, presenting with small, long-lasting macular lesions in the buccal mucosa [5]. In the present case, the patient was 50 years old male. The clinical presentation may be a pigmented swelling, bleeding, increase teeth mobility, pain, delayed healing of extraction sockets, and enlarged regional lymph nodes [8]. These symptoms are nonspecific and may also present in other pigmented oral lesions [4, 5]. Our case was presented with a painless, blackish growth over the central arch of the mandible. There are several pathways responsible for the malignant transformation of normal melanocytes. c-KIT mutation pathway is common and overexpressed in about 80% of mucosal melanoma. While Bioinformatics Resources and Applications Facility (BRAF) protein mutations common in cutaneous melanomas and observed only in <10% cases of mucosal melanomas [1]. Clinically, pigmented oral lesions are evaluated by ABCDEFG (A-Asymmetry, B-Border irregularities, C-Color variation, D-Diameter > 6 mm, E-Elevation, F-Feeling of itch, burn or pain, and G-Growth) [3, 8, 9]. Histological examination is obligatory if an oral pigmented lesion is not benign clinically [8].

In 1995, the Western Society of Teachers of Oral Pathology (WESTOP) Banff workshop recommended to classify the OMM into 1) an in situ pattern, 2) an invasive pattern, and 3) combined pattern of 1 & 2, and they contribute about 15%, 30% and 55% of cases OMM, respectively [1, 4, 8]. The outlined basis for primary OMM is 1) the demonstration of melanoma in the oral mucosa, 2) the presence of junctional activity, and 3) the inability to reveal extra-oral primary melanoma [9]. The diagnosis of OMM can be established confidently by routine hematoxylin and eosin-stained section [3, 8]. Meleti et al. stated that biopsy is required to establish the diagnosis in cases of malignant melanoma of oral mucosa [8]. In the present case, blackish swelling located at the mandible's central arch was clinically suspected for OMM, confirmed by histology. They may show varied morphological appearance like a spindle, epithelioid, or plasmacytoid [3, 8]. These morphological spectra may mimic mesenchymal, epithelial, or neural tumors, especially if the pigment is absent [3, 4, 9]. The immunohistochemistry markers, including HMB-45, Melan A, and protein S-100, are useful to confirm the diagnosis of OMM [1-4, 8]. The present index case showed spindled shaped cells that were immunopositive for HMB-45, CD117, and BRAF. The histopathological and immunohistochemistry (IHC) findings of the present case were concordant with previously reported cases [4, 6, 7, 9].

Meleti et al. suggested that surgery was the mainstay of treatment, supplemented by radiotherapy [8]. The surgical excision involving disease-free margins with or without neck dissection remained the cornerstone for OMM management [1, 2, 6, 9]. Around 10% to 20% relapse rate has been reported after complete surgical removal [1]. Adjuvant therapies, including chemotherapy, radiotherapy, immunomodulators, immunotherapy, and vaccines, have been used for palliative treatment or metastatic melanoma. The influence of these therapies on survival is still uncertain [1, 3, 4, 6, 9]. The OMM is considered as dismally radiosensitive. Postoperative radiotherapy is commonly used in cases having poor prognostic factors such as lymph node or extra-nodal spread of OMM [9]. Radiotherapy is infrequently used as a primary treatment modality in medically compromised or older patients [4]. OMM's prognosis is relatively poor, with five-year survival between 10% to 30% [1, 3]. The tumor thickness >5mm, ulceration, and >10 mitoses per high power field are proposed as independent prognostic factors [1]. The early and rapid diagnosis with treatment may help to enhance the prognosis [4, 6, 7]. Hence, histopathology evaluation will be desired in all suspected oral pigmentations [6]. In this case, radical excision with neck dissection followed by postoperative radiotherapy was given. The patient was free of recurrence until his last visit to the institute. 

## Conclusions

Primary OMM is a very uncommon malignancy with a relatively dismal prognosis. Because of its aggressive nature, all clinicians dealing with pigmented oral mucosal lesions should raise suspicion of OMM in their differential diagnosis, and adequate biopsy from the lesion is mandatory for early diagnosis and therapeutic intervention.
